# Electron Beam Welding of Dissimilar Stainless Steel and Maraging Steel Joints

**DOI:** 10.3390/ma17235769

**Published:** 2024-11-25

**Authors:** Matúš Geľatko, Radoslav Vandžura, František Botko, Michal Hatala

**Affiliations:** Faculty of Manufacturing Technologies, Technical University of Košice with a Seat in Prešov, 080 01 Prešov, Slovakia

**Keywords:** fusion welding, additive manufacturing, selective laser melting, AISI 316, SS 316L, M300

## Abstract

The incorporation of additive manufactured (AM) metal parts to real assemblies is a crucial issue for the increasing of their industrial utilization. The presented research is devoted to the electron beam welding (EBW) of dissimilar steel joints. Dissimilarity is defined by the various types of steel and manufacturing processes used for the creation of specimens. Conventional AISI 316 stainless steel, selective laser melted (SLM) SS 316L stainless steel, and SLM M300 maraging steel were welded at variable parameters in the form of a welding current and a welding velocity. EBW joints were evaluated considering the macroscopic and microscopic characteristics, as well as a reached microhardness. The obtained preliminary results represent important input data for the follow-up experiments focused on the setting of optimal EBW parameters of welding the dissimilar joints including SLM products, with the consideration of their basic macroscopical and microscopical characteristics, mechanical properties, and residual stresses.

## 1. Introduction

Additive manufacturing (AM) is sophisticated, highly innovative and currently well-known technology that brings many positives into the industrial sector, mainly in the form of the freedom in designing the shape of final components [1]. The dimensions of AM parts are still restrictive, as they are defined by the possibilities of 3D printing machine parameters, concretely by their chamber volume. This phenomenon consequently leads to the issue of their incorporation into the real functional assemblies within the connection of AM products with conventional parts (dissimilar joints) and equally their mutual connection [2]. It also includes components created using laser powder bed fusion (LPB-F) technologies working with the input material in the form of a metal powder [3], whereas some practical applications include design of lattice structures that were experimentally verified in the case of numerically modeled L-PBF AlSi10Mg aluminum alloy with promising results [4]. Another similar experiment was conducted on the LPB-F AISI 316L stainless steel, where roughness was evaluated on different configurations of structures [5]. Considering the requirements on the creation of inseparable joints, metal-based materials demand the necessity of applying the welding technologies for their connection [6].

Within recently conducted experiments, more sophisticated welding technologies have been used for the creation of joints composed of AM materials. Tungsten inert gas (TIG) welding was used for the joining of tubes made of SLM (selective laser melting) AISI 316L stainless steel [7]. The experiment showed that higher temperatures have positive influence on the microstructure, due to its increasing homogeneity in the direction of the weld axis, in which the microhardness decreases. Also, porosity was shown to be significantly influential on the mechanical properties of the weld joint. The same technology and similar material were used in the study by Huysmans, S. et al. [8], but within dissimilar joints (conventional 316L and AM 316L), where the corrosion testing is worth mentioning, as it showed that pitting corrosion is situated mainly in the AM material and the heat-affected zone (HAZ). AM AISI 316L was the material of interest in the study [9], where its microstructure and tensile mechanical properties were compared with the same cold-rolled plates after the laser welding process. Defect-free welds were reached with the ferritic–austenitic mode solidification, and a fracture occurred in the weld metal (WM). For dissimilar joints (AISI 316L), input energy of a laser beam is a critical parameter for the weldability of used materials, as was stated in a study by Matilainen, V.P. et al. [10]. During the comparison of TIG and laser welding of AM and conventional aluminum parts (AlSi10Mg) [11], the most significant factor was shown to be the susceptibility of AM components to the creation of porosity, mainly in the case of a TIG welding. The lowest microhardness was reached on the fusion line of TIG weld composed of two AM parts and in the weld metal center of laser weld. The fracture occurred in the mentioned places during tensile tests. The positive influence of electron beam welding (EBW) technology on the final mechanical properties of specimens made of the same material (AlSi10Mg) was observed [12]. Electron beam welding was also used for the joining of LPB-F Inconel 718 specimens, where sufficient penetration depth was reached (proportional to the heat input), with nail-shaped welds of lower hardness in comparison with the base metal (BM) [13]. EBW was one of the technologies applied on the specific SLM CLF-1 material with good joint formation without observed defects [14].

Considering the AISI 316/316L stainless steel, various EBW currents were tested with the objective of evaluating the microstructure and mechanical properties of welds, where fine austenite grains in base metal and fine equiaxed grain in combination with elongated columnar austenite grains in weld metal were observed [15]. Technology showed high potential during the welding of the same material of 160 mm thickness at one-time penetration using the appropriate technological parameters [16]. Residual stresses in EBW 316L joints were analyzed due to the verification of finite element models [17] and also their sensitivity to the beam oscillation, where influenced corrosion properties were taken into account [18]. EBW is also highly effective for the creation of dissimilar joints of various materials, such as titanium alloys (TC4/TA7), where various microstructures were observed in various areas of a weld, with dislocations in a weld metal [19]. The same technology was effective for the welding of dissimilar nickel-based superalloys (GH3039/IC10) in reaching the sufficient joint without any defects, and the microstructure was composed of cellular dendrites and columnar crystals in the weld metal, with the average value of microhardness being 300 HV in this area [20]. 316L stainless steel was part of the dissimilar joint with the RAFM (reduced-activation ferritic/martensitic) steel [21]. The reached microstructure of a weld metal was martensitic with a small amount of a ferrite, whereas the weld metal of two 316L steels (similar joint) was composed of austenite. The heat-affected zone was not observed on the side of the 316L material. Another study evaluated the EBW joint of AISI 316L steel with the Inconel 718 alloy using X-ray tomography [22]. Welding parameters were tested based on the occurrence of porosity and undercuts.

The maraging steel was experimentally tested for welding using the TIG/GTAW (gas tungsten arc welding) method. Concretely, maraging steel grade 250 was welded, and the microstructure was found to change in both the fusion and heat-affected zone due to the high input of heat. The microhardness decreased in the direction of the weld axis, as the consequence of coarsening the grain [23]. Similar hardness development was described in the study [24], where the same technology was used for the maraging steel grade 300 and the embrittlement fracture behaviors with other mechanical properties within HAZ were tested. Laser welding was used for the joints composed of maraging steel 250, in order to create the optimization of welding parameters and numerical simulation, considering tensile strength, hardness, and penetration depth. The observed microstructure was martensitic in the WM and HAZ, with the highest microhardness in the HAZ [25]. Subashini, L. et al. [26] compared the application of the laser-MIG (metal inert gas) hybrid welding and autogenous laser welding on the same material and considered the influence of wire filler metal on microstructure and mechanical properties. A maraging steel was also the main subject of the critical review study focused on the susceptibility of its weldments to the stress corrosion cracking [27]. A dissimilar joint composed of C300 maraging steel and SS 316L stainless steel was reached using a DED (direct energy deposition) method, whereas the microstructure in the fusion zone was without intermetallic phases, and the evaluated mechanical properties of joints were similar to the stainless steel [28]. During the EBW application on maraging steels, WMs are represented by a dendritic microstructure [29], with the presence of a certain HAZ with microstructure similar to BM and the observation of a dark band region [30].

The experiment described is devoted to the application of EBW technology for the creation of dissimilar steel joints, whereas a dissimilarity is represented by both different manufacturing technology of base metals (SLM and conventional) and different steel kinds (stainless and maraging). The motivation of the research comes out directly from the unusual combination of the mentioned base metals, their production technologies, and obviously the welding technology, within which it is necessary to find the appropriate technological parameters for the creation of sufficient joints. Within this first stage of research, welds are evaluated from the macroscopic and microscopic view, and microhardness is measured, all based on various parameters in the form of a welding current and a welding velocity. The results are a necessary steppingstone for the comprehensive research, focused on the evaluation of created EBW welds of dissimilar SLM materials, with the objective of sufficient incorporation of metal AM components to greater assemblies, such as demanding engineering parts or specific cutting tools.

## 2. Materials and Methods

Three materials were selected as the experimental specimens: conventional stainless steel AISI 316, AM stainless steel SS 316L, and AM maraging steel M300. The following Table 1, Table 2 and Table 3 include the chemical compositions of the mentioned base metals. The main physical, mechanical, and thermal properties of the experimental materials are included in Table 4.

Experimental specimens of SS 316L and M300 materials were prepared using selective laser melting (SLM) technology. Specimens were prepared in the Technical University in Ostrava (Ostrava, Czech Republic)–Center of 3D printing Protolab. For SS 316L stainless steel and for M300 maraging steel, RenAM500S Flex and AM400 systems by Renishaw company (Wotton-under-Edge, UK) were used, respectively. Both machines provide production speed at 5–20 cm^3^·h^−1^, scanning speed up to 2000 mm·s^−1^, positioning speed up to 7000 mm·s^−1^, and layer thickness between 20 and 100 µm. The first can create parts with laser power up to 500 W and the second up to 400 W in chambers of 250 × 250 × 350 mm and 250 × 250 × 300 mm, respectively. Particle distribution for both powders is in the range of 15–45 µm. Technological parameters for SLM preparation of experimental materials are included in Table 5.

*ε* = *P*/(*v* × *d* × *t_L_*) (1)

**Table 5:** SLM technological parameters.

Parameter	SS 316L	M300
Laser power *P* [W]	200	400
Scanning velocity *v* [mm·s^−1^]	650	820
Hatch distance *d* [μm]	110	95
Layer thickness *t_L_* [μm]	50	40
Energy input [J·mm^−3^] (1) [34]	55.94	128.37
Scanning strategy (Figure 1)	Chessboard	Meander
Gas protection	Argon (Ar)	

**Figure 1:**
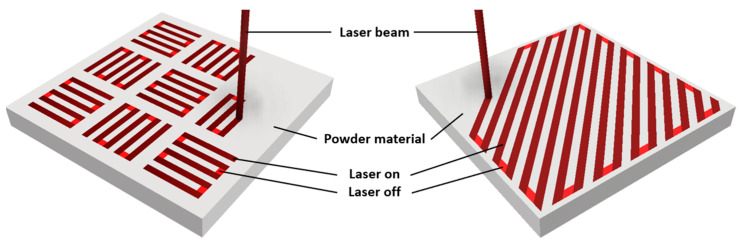
Stripe (**left**) and meander (**right**) scanning strategies [35].

Experimental materials (50 mm long, 20 mm wide, and 10 mm thick) were joined by the fusion welding using the EBW technology that uses a high concentration of electrons arising in the focal point of the electron beam, which causes local heating and the melting of influenced materials. Welds were created in First Welding Company, Inc., in Bratislava (Slovakia) using the automated EBW device PZ ELZA UNI 2G, with a vacuum chamber (10^−2^ Pa) in dimensions of 1300 × 1500 × 1550 mm and motion range up to 451 mm on the X and Y axes. Accelerating voltage was between 25 and 60 kV, electron beam power was in the range of 30 W to 30 kW, and the range of the welding current was 1–500 mA. The characteristic EBW weld bead profile of the experimental specimens was reached by the influence of the electron beam and its motion in a longitudinal direction of welded specimens, when the keyhole was created during the melting, which defines the weld bead profile after solidification. Its shape was also influenced by the EBW technological parameters. The following Table 6 summarizes the parameters of welding the experimental specimens, which were divided into three groups. The first group includes mutual joints of conventional AISI 316 steels for adjusting the main welding parameters. The second group includes joints of AISI 316 and SLM SS 316L steels for the evaluation of welding the stainless steels with different manufacturing processes. The third group includes joints of AISI 316 and SLM M300 steels for the evaluation of possibilities in welding of dissimilar joints composed of various steels, considering different manufacturing technologies and compositions. All specimens were welded using accelerating voltage (U_a_) at 55 kV, focusing current (I_f_) at 833 mA, and the oscillation diameter of 1 mm at 555 Hz frequency (f_o_).

Welded specimens were subjected to the observation of macrostructure and microstructure in the cross-section due to the evaluation of morphologic geometry of the weld joint; rate of metallographic fusion of base metals; and characteristics of WM, HAZ, and BM. The prepared metallographic specimens were macroscopically analyzed using the Stemi 508 stereo microscope with an AxioCam ERc 5s camera (Carl Zeiss AG, Oberkochen, Germany). Microscopic analysis was conducted using the Axioskop 2 MAT optical microscope (Carl Zeiss AG, Oberkochen, Germany) with an AxioCam ERc 5s camera and a Keyence VHX-7000 digital microscope (Keyence, Osaka, Japan). The measurement of microhardness was conducted using the Micro-Vickers Hardness Tester CV-400DAT (CV Instruments, Borgharenweg, The Netherlands) at 2.942 N force and dwell time for 15 s.

## 3. Results

### 3.1. Macroscopic Evaluation

**Figure 2:**
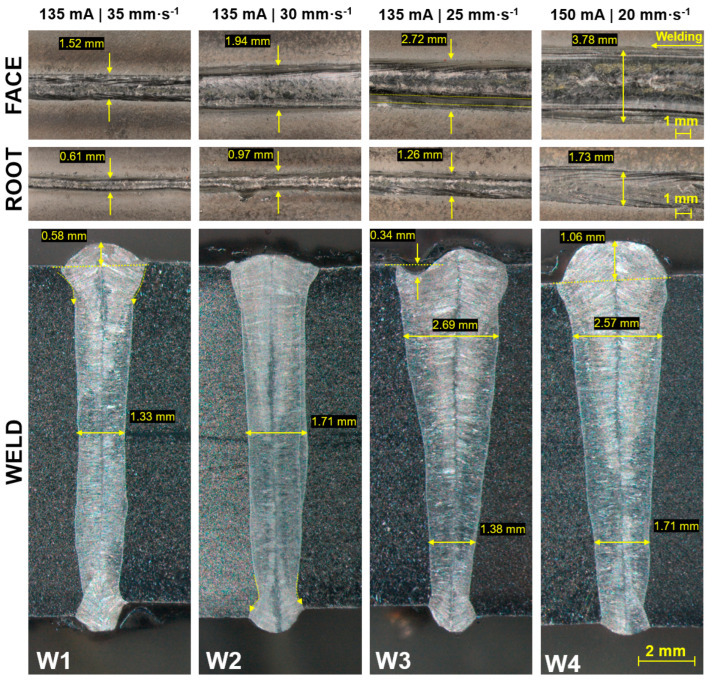
Macroscopic view of AISI 316–AISI 316 welds.

In order to adjust EBW parameters for experimental specimens, four welds were created of conventional AISI 316 stainless steels (Figure 2). All these joints included typical characteristics of the EBW process with certain discrepancies. The first weld (W1) was made using the 135 mA current at 35 mm·s^−1^ velocity. The whole thickness of base metals was sufficiently melted and fused in the form of a nail-shaped joint. However, the width (1.33 mm) of the weld joint was not quite auspicious. The steep narrowing right below the reinforcement was observed, as the consequence of the combination of the lower welding current with higher velocity. This phenomenon was suppressed by lowering the velocity to 30 mm·s^−1^ (W2). The nail joint was thicker with stable dimensions throughout the whole depth. Also, the excess of reinforcement was suppressed. In this case, a steep transition into the root area was observed. A wedge-shaped weld (W3) was reached at lowering the velocity to 25 mm·s^−1^, with the thickness of 2.69 mm below the reinforcement and 1.38 mm above the root, whereas its shape was smooth through the whole depth without any steep transitions. A significant discontinuity was observed in the form of an undercut on the left side of the face. The last weld (W4) of the first group was created using the higher current (150 mA) and a velocity of 20 mm·s^−1^. In comparison with the W3 weld, the reached width was higher, mainly in the area above the root (1.71). The reached width of the face was 3.78 mm, and the root was 1.73 mm wide; hence, the typical texture of a weld in a welding direction was visible. It is worth mentioning the significant excess of reinforcement, which was probably caused by the combination of a higher welding current and lower welding velocity.

**Figure 3:**
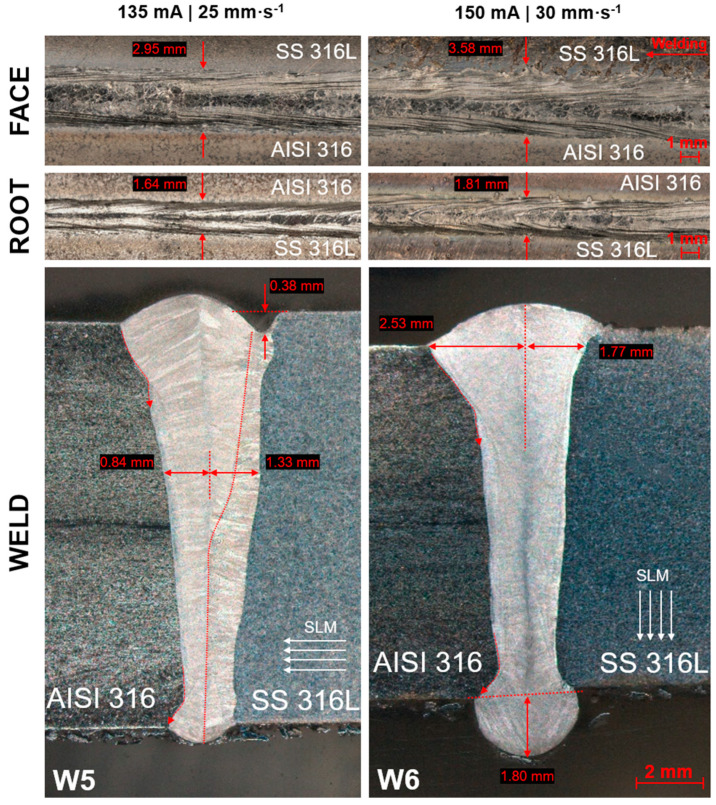
Macroscopic view of AISI 316–SS 316L welds.

Similar welding parameters were used for the creation of dissimilar joints within the second group (Figure 3). For the first weld (W5) of this group, the same parameters of W3 weld were used (135 mA, 25 mm·s^−1^). The reached basic shape characteristics of the wedge joint were also similar to the W3 weld, with the sufficient thickness of reinforcement and sequential narrowing in a direction to the root. A dissimilarity of base metals (different manufacturing processes) caused various dimensions of the weld to interface to its center axis, which was longer on the side of the SLM material in this case (1.33 mm). Also, the undercut was observed on this side, as well as the sudden variation of weld metal texture in the area of the highlighted curve, which could have been the consequence of the mixing of liquid metals in the fusion zone. On the other side of the weld (conventional base metal), a steep transition was observed in both below the reinforcement and above the root. For the W6 weld, the same current (150 mA) was used as in the case of the W4 weld, but the velocity was set at 30 mm·s^−1^, due to the excess of reinforcement on the W4 weld. The weld metal was fused to a sufficient extent. The phenomenon of various weld dimensions to its center axis was not present through the whole depth of the wedge joint but only at the area below the reinforcement. In comparison with the W5 weld, a higher input energy caused a more significant impact of the electron beam, which resulted in the non-negligible root dropout in the evaluated cut of a weld.

**Figure 4:**
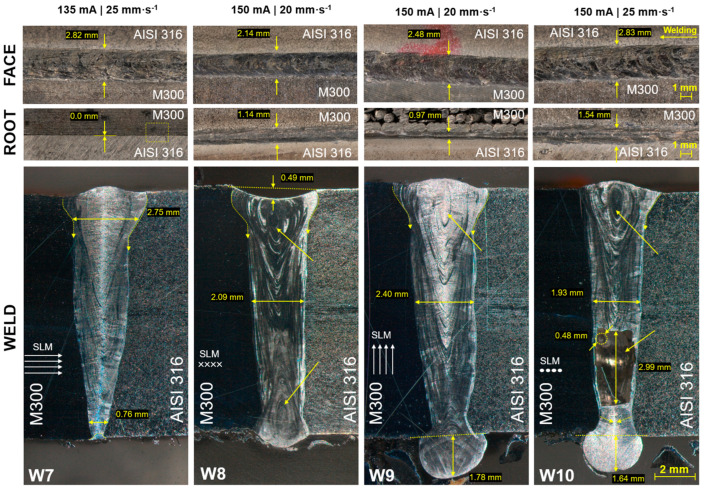
Macroscopic view of M300–AISI 316 welds.

Using the welding current at 135 mA and welding velocity at 25 mm·s^−1^ was also verified for the creation of the W7 weld (Figure 4). It is evident from the shape of the joint that the impact of the electron beam at these parameters was insufficient. The upper part of this wedge weld fulfils basic requirements. However, low welding parameters caused steep transition of its width below the reinforcement on both sides and a significant narrowing of joint, with the insufficient fusion of base metals on the root surface. Increasing the current to 150 mA and decreasing the velocity to 20 mm·s^−1^ caused the improvement in the weld thickness uniformity in a direction of depth (2.09 mm) within the straight W8 weld with the sufficient creation of weld root (1.14 mm wide). Similarly, steep transitions below the reinforcement were present, with a considerable underfill of the weld face. Vortex textures, caused by the formation of keyhole due to the sufficient energy density, were observed on both sides of a depth direction. The upper vortex is the consequence of the molten metal flow under the influence of surface tension, and the lower vortex is the consequence of the influence of the electron beam and the bottom surface tension. Within previously described welds (W1–W7), molten metal flows upward around the weld axis, caused by the recoil pressure arising during the metal vaporization and the flow on the surface of the weld driven by the hydrostatic pressure arising during the motion of the weld beam [36]. The energy input was lower, and hence the flows of molten metal below the upper surface and above the bottom surface were not present. The application of the selected EBW parameters on the W9 weld caused the creation of a straight weld with similar thickness (2.40 mm). However, the reinforcement highness is satisfactory, but the root dropout occurred in this case. The root width was stated at 0.97 mm, but the view was distorted due to the excess of SLM support structures. Increasing the velocity (W10 weld) caused the creation of a joint with similar characteristics. Differently, the weld thickness was smaller in the place of the cut, and the epitaxial growth of the substructure composed of columnar crystals was observed above the root area; thus, the metal flow above the root surface was not present due to the insufficient impact of the electron beam in a deeper volume of the weld. However, face and root width were greater (2.83 mm and 1.54 mm, respectively). A serious defect was observed in the lower part of the weld through its whole thickness. This cavity seemed to be the consequence of higher speed and consequently lower energy density that led to insufficient time for solidification that could not stand the impact of cutting during the preparation of specimens for the microstructure analysis. Another source can be the porosity cluster, which caused a division of larger amount of material after its yield to cutting during the preparation of specimens. A residual pit after the pore presence is highlighted in the left upper corner of the cavity. Such porosity could be transferred from base metals, mainly M300, which could be porous after the SLM process.

**Figure 5:**
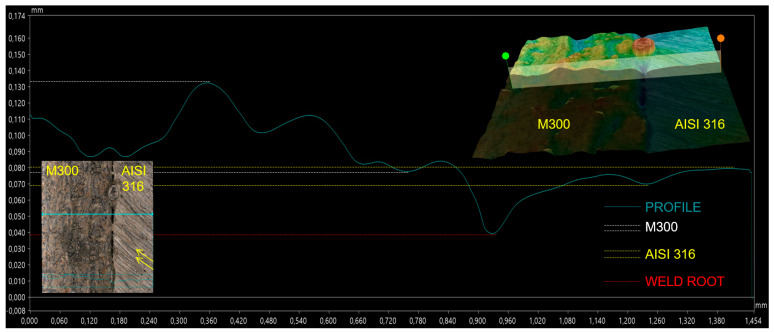
Profile of the root area on the W7 weld—insufficient fusion.

The parameters used for the welding of the W7 specimen caused insufficient root penetration. Detailed analysis on the bottom surface of a specimen showed that the root penetration was different along the length of the weld (highlighted area of the root in Figure 4). The following figures depict various profiles within this area. Figure 5 includes the profile of the weld joint, where base metals were not fused on the bottom surface. The created crater was in the recorded depth around the level of 0.040 mm, which was approximately 0.035 mm below the analyzed surface. It is worth mentioning that surface profiles of base metals are different, mainly due to the various manufacturing processes. The profile of AISI 316 stainless steel was smoother within the evaluated area. A texture is defined by the traces in a diagonal direction as the consequence of the cutting process. The base metal of the M300 maraging steel was more articulated without a defined pattern, caused by the higher roughness reached after the SLM process. The profile in the evaluated area included more deviations.

**Figure 6:**
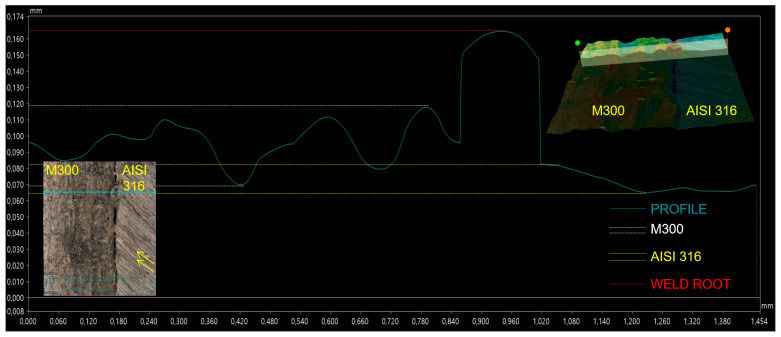
Profile of the root area on the W7 weld—root dropout.

The following Figure 6 includes the profile on the line where root dropout occurred. Similarly, the profile on the side of the AISI 316 base metal was smoother with negligible deviations. On the side of the M300 base metal, variations of profile were more significant, but in comparison with the area in the previous Figure 5, the range was smaller. In this case, the peak was reached on the weld center axis, where the mentioned root dropout was present. Besides the fact that the used welding parameters were not able to fuse base metals on the bottom surface, the molten metal reached the root in some points. The absence of solidified material around this point on the weld axis caused an overflow of the mentioned molten metal, which caused the root dropout after the solidification.

**Figure 7:**
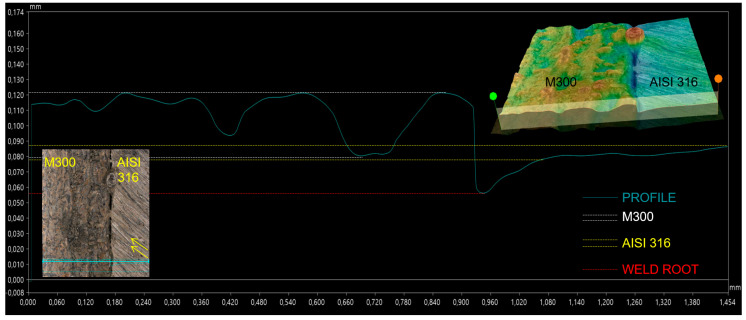
Profile of the root area on the W7 weld—insufficient fusion.

The profile depicted in Figure 7 includes the previously mentioned characteristics of conventional stainless steel (AISI 316) and SLM maraging steel (M300). However, the variance of deviations was the lowest in comparison with the other evaluated lines (0.08–1.120 mm). Furthermore, base metals seemed to be fused from the view on the bottom of the weld, but the profile curve depicts a certain valley on the center axis of the weld, and hence it can be stated that the used welding parameters were highly insufficient for these experimental specimens.

### 3.2. Microscopic Evaluation

**Figure 8:**
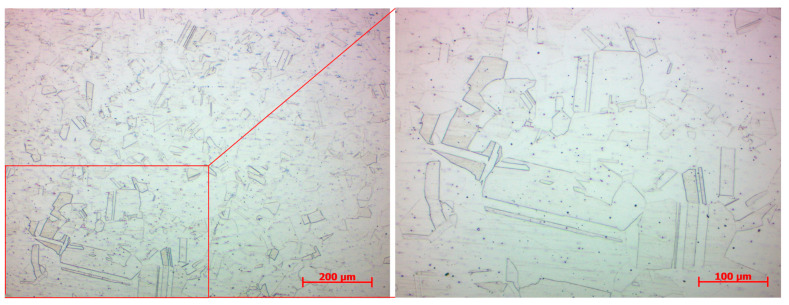
Microstructure of the conventional AISI 316 base metal (W3 specimen).

The microstructure of the conventionally made base metal (AISI 316) within welds was polyhedral–characteristic for stainless steels, including austenitic grains with equiaxed, variously oriented shapes sized between 10 and 100 µm (Figure 8). The shape morphology of grains stayed unchanged after the welding with the occurrence of annealing twins. The matrix also included unorganized dispersing of morphologically heterogeneous precipitates of alloying elements’ (Cr, Ni, etc.) carbide phases. Any discontinuities or defects, such as porosity or metallurgical inclusions, were not identified during the observation of AISI 316 base metal microstructure.

**Figure 9:**
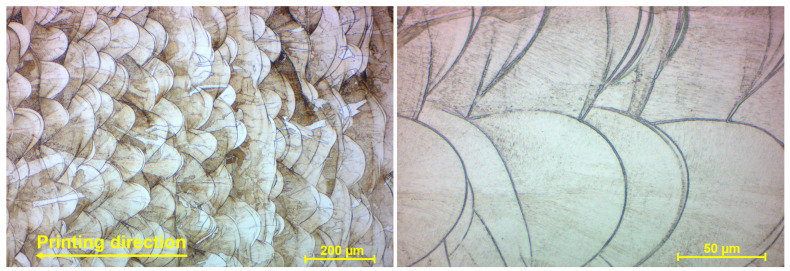
Microstructure of the SLM SS 316L base metal (W5 specimen).

The microstructure of SLM SS 316L base metal is characterized by the spherical substructure of grains representing the solidified melt pools, defined by a technological process, during which the laser beam impulses melt a material layer by layer (Figure 9). The size of grains is approximately 100 µm in a perpendicular (laser motion) direction and 20–100 µm in a printing (layering) direction, whereas this range is defined by the penetration of individual melt pools and their mutual interaction and bonding during the layering process. The structure of grains itself, growing in the direction of the layering process, is dendritic and identical with a direction of heat flow during the solidification. A discrete and heterogeneous segregation was observed on the grain boundaries, copying the shape of the substructure, due to the faster solidification, and hence more rapid recrystallization. In comparison with conventional material, precipitates of carbide phases of steel were not monitored in the matrix, which can be ascribed to the causal impact of SLM technology.

**Figure 10:**
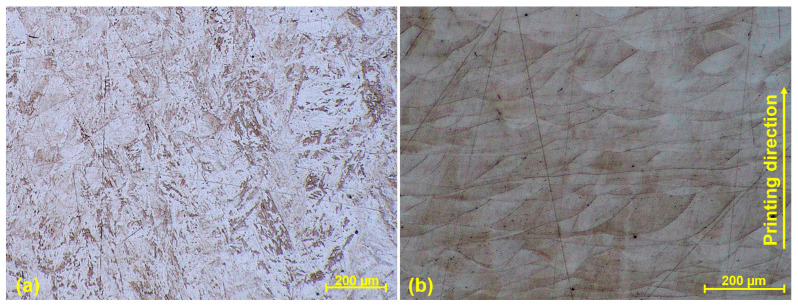
Microstructure of the SLM M300 base metal (W9 specimen); (**a**) lath martensite single-phase microstructure; (**b**) dendritic structure (SLM melt pools).

The base metal represented by the SLM M300 maraging steel exhibited a typical lath martensite single-phase microstructure (Figure 10a). The melt pools created during the SLM process were not so expressive, being adjusted using a different etchant (Figure 10b). The spherical substructure of grains was more evident, and individual printing layers could be distinguished. In the perpendicular direction, the size of grains was approximately 100–150 µm, and the size in the printing direction was mostly approximately 10–50 µm. Similarly to the SLM SS 316L material, the range was defined by the overlapping of layers, and hence a smaller range in the printing direction was the consequence of smaller layer thickness. The grains were composed of a dendritic structure, with the growing trend and solidification in the direction of the heat flow. A segregation was present on melt pool boundaries by the same previously mentioned reason. Certain discrepancies were observed in the SLM M300 base metal, in the form of black dots. It can be the individual and randomly occurred porosity, which arises under the influence of gas entrapped in the molten material and stays as a gap in the solidified material.

**Figure 11:**
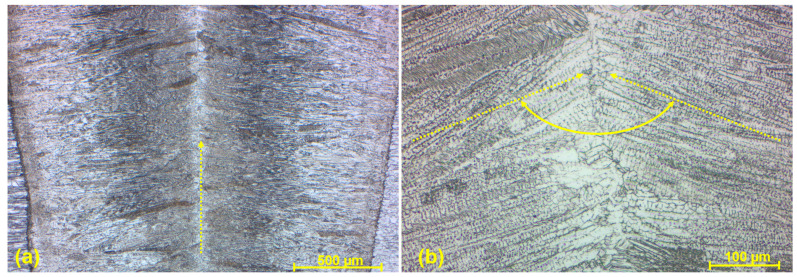
Microstructure of the AISI 316–AISI 316 weld metal (W3 specimen); (**a**) WM with epitaxial growth of substructure;(**b**) columnar crystals oriented at dihedral angle.

**Figure 12:**
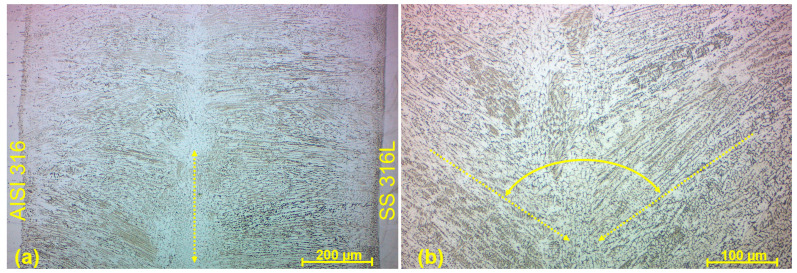
Microstructure of the AISI 316–SS 316L weld metal (W5 specimen); (**a**) WM with epitaxial growth of substructure; (**b**) columnar crystals oriented at dihedral angle.

A weld joint of two AISI 316 stainless steels (Figure 11a) was fully fused with base metals throughout the whole thickness of a single created welding layer. Its microstructure was mainly dendritic as a consequence of rapid heat removal during the solidification of liquid weld metal after the EBW process, with the epitaxial growth of substructure in the form of columnar crystals mutually oriented at a dihedral angle (Figure 11b). In addition was the influence of the previously described molten metal flow up in the direction of the weld axis in combination with the decrease in solidification speed in a direction from the BM to the WM. Furthermore, a microstructure is composed of cellular crystals and equiaxed grains present in the center of the weld metal without the significant segregation or drains. A finer microstructure is the consequence of the EBW characteristics, where a thin fusion zone and subsequent more rapid solidification does not cause the creation of such coarsened grains. The same description of WM microstructure can be applied for a dissimilar weld composed of AISI 316 and SLM SS 316L (Figure 12), reporting similar characteristics. However, the angle of columnar crystals was the opposite due to the analyzed area closer to the lower part of the weld joint, where they tended to direct towards the root surface. Considering the frequently occurring defects, no cracks, cavities, pores, inclusions, or incomplete fusion with base metals were observed in these two analyzed joints.

**Figure 13:**
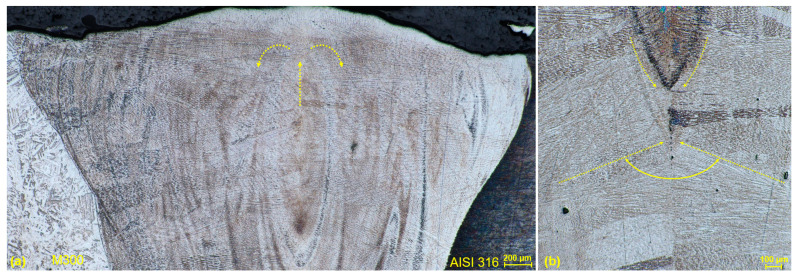
Microstructure of the M300–AISI 316 weld metal (W9 specimen); (**a**) vortex shape in the upper part of WM; (**b**) tip of the vortex with columnar crystals oriented at dihedral angle.

The weld composed of AISI 316 stainless steel and M300 maraging steel was created in a sufficient fusion of both metals without the observation of a significant lack of material mixture and related weld defects. A fine dendritic microstructure was similarly created. Columnar crystals were mostly oriented perpendicularly to the weld axis. However, the texture of WM in the upper part of the weld was notably influenced by the molten metal flow in combination with the presence of surface tension, resulting in the vortex shape (Figure 13a). A typical epitaxial growth of a substructure (columnar crystals) was observed right below the tip of the vortex (Figure 13b). Characteristically for the EBW, the fine microstructure reached was caused by a thin fusion zone and fast solidification, no matter what kind of steels were welded. The fusion zone exhibited darker zones on the left side of the WM, being influenced by the higher presence of martensite on the side of the M300 material. The etching adapted to the maraging steel also influenced this phenomenon and caused the blackening of AISI 316 BM. The observed discontinuities in the form of porosity were related to those described in M300 BM, which could be transferred to the weld metal. Any cracks or incomplete fusion with BM were not observed in this joint.

**Figure 14:**
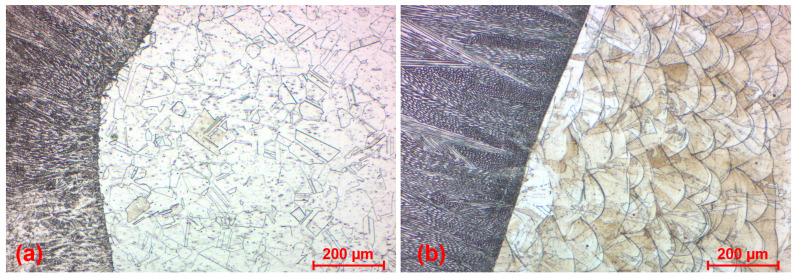
Heat-affected zone: (**a**) AISI 316 (W3 specimen); (**b**) SS 316L (W5 specimen).

The heat-affected zone of conventional stainless steel (AISI 316) was composed of a microstructure identical to the base metal, with the rapid transformation to the weld microstructure on their boundary (Figure 14a). Any recrystallization of the austenitic matrix was not observed, wherein carbide phases precipitates were structurally and morphologically retained. Similarly, the heat-affected zone of SLM stainless steel (SS 316L) resembled typical spherical-shaped grains of a base metal up to the weld boundary, with the sufficient fusion of the weld metal and a fluent transition (Figure 14b). Hence, any morphological transformation did not occur after the solidification process within HAZ. From the side of the weld metal, WM microstructure, represented by the columnar crystals, was present right after the boundary in both cases. Such heat-affected zones are characteristic for the EBW process, where the energy of beam penetrates more significantly into the depth of a material than into the weld width direction. Also, the quite rapid solidification plays its role when there is not enough time for the gradual material recrystallization between WM and BM.

**Figure 15:**
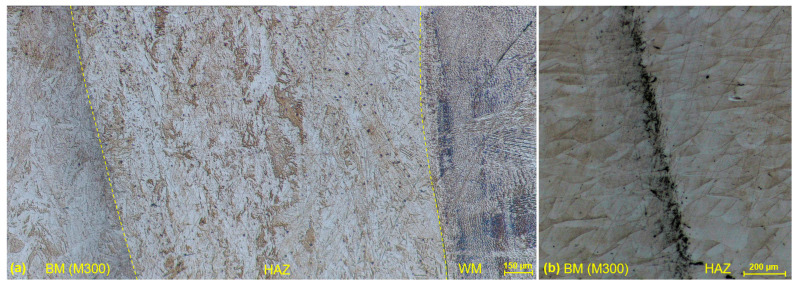
Heat-affected zone of the M300–AISI 316 weld (W9 specimen); (**a**) lath martensite single-phase microstructure in HAZ; (**b**) dark band zone with SLM melt pools.

In comparison with previous negligible HAZs, the one of the M300–AISI 316 weld was noticeable throughout the whole thickness of the joint (approximately 1.5 mm). The etching was adapted to the depiction of all three microstructures (WM, HAZ, and BM) at a sufficient level (Figure 15a). The finer lath martensite single-phase microstructure of BM was unchanged during the transition of material through the HAZ, up to the WM, where a dendritic microstructure was present right after the fusion line. From the side of the BM, the HAZ was bounded with a dark band zone, being closer to the line character (approximately 150 µm wide). A dark band zone is represented by the two-phase microstructure, composed of martensite and austenite. The reverted austenite is created in a martensite matrix due to the influence of high temperatures on martensite stage [25]. After the etching for a depiction of the characteristic SLM melt pools (Figure 15b), a dark band zone is a more significant, and an unchanged spherical microstructure of a BM in a HAZ is more evident. The previously described porosity observed in M300 BM and WM were also present in the HAZ.

### 3.3. Microhardness Measurement

**Figure 16:**
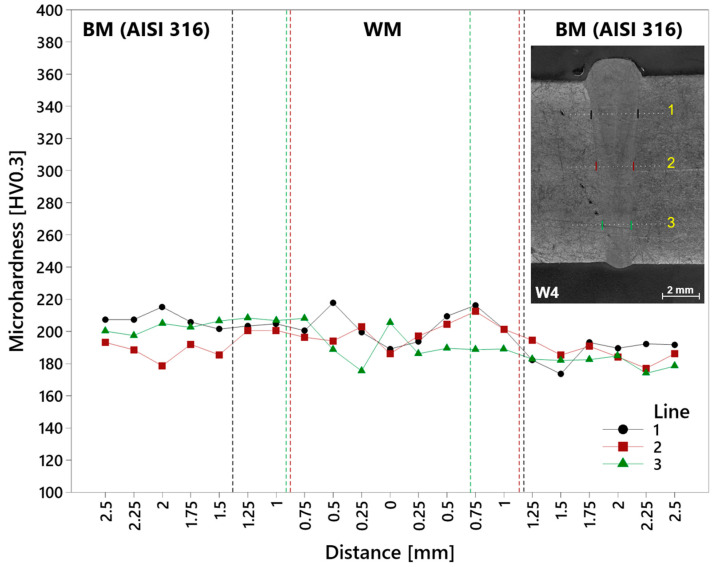
Microhardness of the AISI 316–AISI 316 weld (W4 specimen). Fusion lines (dashed): black—Line 1; red—Line 2; green—Line 3.

Three specimens (one of each group) were selected for the microhardness measurement, and each specimen was measured in three lines composed of 21 impressions, by which the reaching of sufficient distance in base metal from the weld was secured. The zero point within each line was in the center of the weld metal, and 10 impressions were made in both directions of base metals, with their mutual distance of 0.25 mm. The mutual distance between lines was 3 mm and was 2 mm from the upper and lower surfaces. The graphs do not include HAZ regions due to their absence (minimal size) at the EBW welds. The dividing lines of weld regions are highlighted separately for all three measurement lines due to the irregular width and shape of the weld in a depth direction. The following Figure 16 includes the microhardness of the W4 specimen. The microhardness of the weld can be considered as being consistent in the evaluated area of measurement lines, with the values around a BM value. Overall, the measured microhardness values were defined by the austenitic microstructure of the BM and a fine microstructure of the WM. The upper part of the weld (Line 1) reached 189 HV in the center with its increasing in both directions and a stabilization in both base metals. Line 2 from the middle part of the weld showed similar microhardness in the center of the weld (186.1 HV), with a similar increasing and stabilization trend. Microhardness within the lower part of the weld (Line 3) reached a higher value in the center (205.3 HV), with a decrease in both directions before stabilization. On the right side, stabilization occurred already in the weld metal.

**Figure 17:**
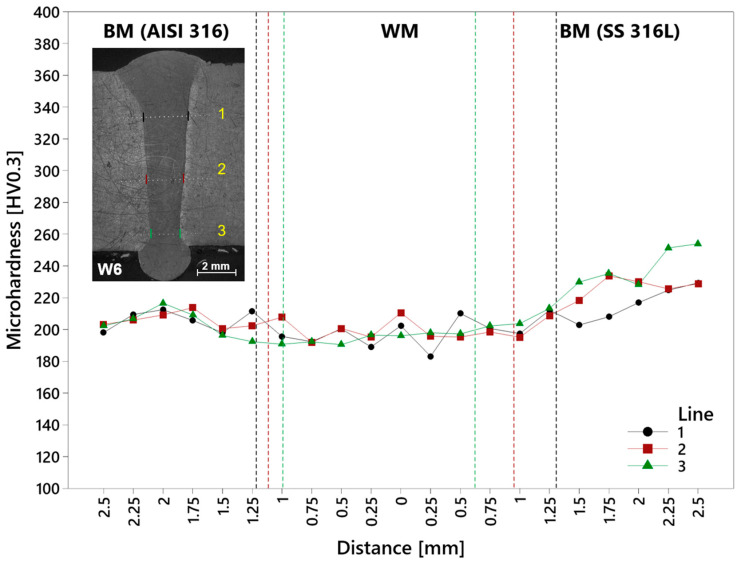
Microhardness of the AISI 316–SS 316L weld (W6 specimen). Fusion lines (dashed): black—Line 1; red—Line 2; green—Line 3.

The lines of a microhardness measurement on the W6 specimen were situated lower from the upper surface, due to the not negligible width of a reinforcement (Figure 17). The overall microhardness of a weld is on the level of conventionally made AISI 316 stainless steel with the increase on the side of the SLM SS 316L stainless steel, a little higher than a data sheet value. Nevertheless, the AISI 316 stainless steel included higher carbon content, which caused the increasing of microhardness, and the SS 316L material reached higher values. It is the consequence of the SLM process, which creates finer austenitic microstructure with dislocations of higher density situated on grain boundaries on the substructure level, which lead to the higher resistance of material to the indentation [37,38]. The upper part (Line 1—below the reinforcement) reached 202.3 HV in the center of the weld with the following decrease in both directions. In the direction of the AISI 316 material, values were stable. In the opposite direction, the gradual increase to the maximum occurred in the BM (229.3 HV). The microhardness of the middle part (Line 2) progressed with a similar trend, where the 210.5 HV in the center of the weld decreased to lower values with a stabilization in the direction of conventional base metal. In the direction of the SLM material, the increasing of stable values started right after the weld boundary. The overall maximum of the weld was reached in the SLM material on Line 3 (253.9 HV). The microhardness in a weld metal is the most stable in this area.

**Figure 18:**
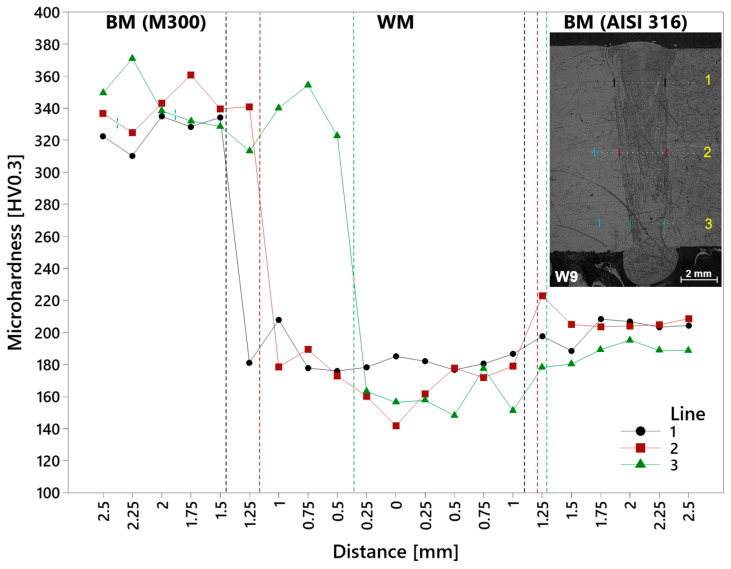
Microhardness of the M300–AISI 316 weld (W9 specimen). Fusion lines (dashed): black—Line 1; red—Line 2; green—Line 3; blue—HAZ (BM M300).

The microhardness of the specimen W9 was not so consistently distributed in the evaluated area of the weld (Figure 18). The curves represent minimal values in the area of a weld metal, caused by the mixing of different base metals of various compositions in a fusion zone. The slight increase was recorded in the direction of AISI 316 stainless steel and significantly in the direction of SLM M300 maraging steel, approximately to the level of data sheet values. The high microhardness of a maraging steel is provided by its specific chemical composition, even though the natural hardness-increasing carbon (C) content is low (≤0.03). The final martensite microstructure is generally characterized by higher hardness, whereas the important factor is higher nickel (Ni) content, which suppresses the creation of ferrite and pearlite, as well as cobalt (Co), increasing the hardness to high values through the acceleration of precipitation and subsequently reducing the duration of ageing [39]. Furthermore, higher titanium (Ti) content also has the ability to increase the hardness of maraging steel [40], which can be ascribed to the influence of higher dislocation density and intergranular zones including Ti and precipitates of CoNi [41]. Worth mentioning are the non-negligible deviations, mainly in the areas of weld metal and M300 base metal. The upper part of the weld (Line 1) reached 185 HV in its center, which can be considered stable in the whole weld metal. Right beyond the border, microhardness rapidly increased. On the other side of the weld, a slight increase in microhardness occurred in the AISI 316 base metal, which stayed stable in points situated further from the weld. The overall minimum of the whole weld was reached in the zero point of the middle part (Line 2) of the weld (141.8 HV). Microhardness increased in both directions, slightly towards the AISI 316 material with its stabilization in a base metal and significantly on the boundary of the weld with the M300 base metal. A rapid increase was observed in the lower area of the weld (Line 3), where the second (0.5 mm) point was impressed in the M300 HAZ due to the shape of the weld joint. The same as in the case of points within other lines, the slight increase occurred in the direction of AISI 316 stainless steel with a stable state in a base metal. A difference in microhardness between a HAZ and a M300 BM was not observed due to the unchanged microstructure. The boundary line represented by a dark band zone is highlighted with blue lines in Figure 18.

## 4. Discussion

The described characteristics of welds within this study are in a certain correlation with other studies from the field. The used welding parameters (adjusted mainly according to similar AISI 316 welds) were different from other studies [15,16,18] at first sight. However, considering the various thicknesses of experimental specimens within the mentioned experiments, the used parameters can be regarded as similar. Considering the macroscopical parameters of the reached joints, the fusion zones of welds in all three groups were significantly larger in a depth direction than in the width direction, which is characteristic for EBW, no matter what the thickness of the weld is, having been proven in the case of 160 mm welds with the large weld depth–width ratio [16]. The overall shapes of welds were nail- and wedge-shaped, similarly stated in the study [17] (wine-glass shape) and the study [21], where also no significant weld defects were observed and HAZ was negligible in the case of AISI 316L stainless steel joints. Additionally, the same shape characteristics were observed within dissimilar AISI 316–SS 316L and M300–AISI 316 welds in this experiment. The microstructure of stainless steel welds (similar and dissimilar) was transformed from the austenitic equiaxed grains in the BM to the columnar and partly equiaxed grains in the WM right after the fusion line without the significant HAZ, which corresponds to results of related studies [15,16,21]. In the case of the SS 316L material, the same transition of the microstructure in the direction of the weld axis was observed, despite the fact that the BM austenitic microstructure is influenced by the SLM technology. The martensitic microstructure of M300 BM was retained within the observed HAZ, and a dendritic microstructure was present in the WM, which was also described in the case of other studies focused on the EBW welding of maraging steels [29,30]. HAZ is divided with a BM by a dark band zone, being thinner in comparison with other welding methods [30], whereas it was shown to also be related to the etching of the analyzed specimens. The HAZ of the AISI 316 material was also not present within a dissimilar weld with the M300 material. A microhardness in all welds was on the level of the ASI 316 base metal, which was also described in other studies [15,21], with the exception of [16], where a little increase was recorded in the weld center (15–20 HV higher than BM). Within dissimilar joints, the microhardness increased in the direction of the SS 316L base metal, which is the consequence of a different manufacturing technology (SLM), and a significant increase was recorded in the direction of the M300 base metal, whereas the presence of M300 HAZ did not have an influence on the microhardness of a joint. A similar distribution of microhardness in similar materials was also described by using the other welding technologies, such as TIG welding of SLM stainless steel [7] or GTAW/TIG welding [23] and laser welding of maraging steels [25]. Additionally is a depiction of microhardness distribution within dissimilar EBW joints, including SLM SS 316L stainless steel and SLM M300 maraging steel, within the presented study. A description of characteristics of dissimilar joints including unusual used materials representing stainless steels (AISI 316 and SLM SS 316L) and mainly SLM M300 maraging steel expresses the overall added value of a described experiment.

## 5. Conclusions

The presented experiment focuses on the evaluation of possibilities in the electron beam welding of dissimilar welds composed of different types of steels that were produced using different manufacturing processes. Three groups of specimens were divided according to welded base metals, wherein the first included two conventional AISI 316 stainless steels, the second included conventional AISI 316 with SLM SS 316L stainless steels, and the third included M300 maraging steel with conventional AISI 316. The created welds, reaching variable parameters (welding current and welding velocity), were evaluated regarding three main aspects (macrostructure, microstructure, and microhardness), based on which the following statements can be concluded:

- The selected EBW parameters were able to reach sufficient weld joints in all three groups of materials without the significant lack of fusion. The combination of the 135 mA current and the velocity in the range of 25–35 mm·s^−1^ was appropriate for the creation of sufficient joints within the first and second group of specimens, and hence it can be stated that materials on the similar base (316 stainless steel) can be welded with the same parameters. The current of 150 mA was suitable for both groups and more appropriate for the third group of specimens using a certain velocity. The influence of SLM orientation on the creation of welds was not recorded.
- Within the first group, the application of lower current and higher velocity created nail-shaped joints, whereas a decrease in velocity caused the reaching of wedge-shaped joints, which also applies for the second group. The same effect on the shape of a joint was reached using the higher welding current. Appropriate lower-current parameters (135 mA and 25 mm·s^−1^) for stainless steels were not sufficient for the third group joints, where the lack of root penetration occurred. A 150 mA current with the appropriate velocity (20 and 30 mm·s^−1^) created straight welds of a sufficient width and the presence of a vortex, acting as an indicator of good molten metal flow. From macroscopical defects, excesses of reinforcement, undercuts, root dropouts, underfill of the face, and a cavity were observed.
- The microstructure of AISI 316 BM was austenitic with equiaxed grains and annealing twins. In the case of the SS 316L BM, a microstructure was defined by the spherical grains with dendritic substructure, as the consequence of the SLM process. Within the M300 BM, lath martensite single-phase microstructure and a spherical microstructure with a dendritic substructure were observed, according to applied etchant. The microstructure of WM in all three groups was mainly dendritic with the epitaxial growth of substructure in the form of columnar crystals. In the case of the third group, the texture was influenced by the molten metal flow and the creation of a vortex. The excessive HAZ was only present in the third group with a thin dark band zone (M300), without significant recrystallization. Discontinuities were observed in M300 BM and their welds.
- The microhardness of WM was on the level of AISI 316 BM in all three analyzed welds, whereas it increased in the direction of SS 316L BM due to the finer microstructure and more significantly in the direction of M300 BM for the same reason, whereas the microhardness in the HAZ was unchanged due its similar microstructure with the BM. Furthermore, it must be mentioned that it is appropriate to adapt the number of measuring points to the width of the present HAZ.

According to the examined aspects, it can be stated that AISI 316 stainless steel has the potential to be a satisfactory base metal for the connection of SLM SS 316L and mainly SLM M300 maraging steel with subassemblies. However, it is necessary to conduct subsequent experiments focused on the evaluation of other mechanical properties. The application of scanning electron microscopy can be helpful for better microstructural analysis, or X-ray diffraction for the evaluation of residual stresses, which has an important influence on weld. Considering the selection of EBW parameters, the design of experiment (DoE) method with follow-up analysis could bring a better understanding of their influence on the evaluated characteristics of weld joints.

## Figures and Tables

**Table 1:** Chemical composition of AISI 316 stainless steel [31].

Element	Fe	Cr	Ni	Mo	Mn	Si	N	P	C	S
Mass (%)	Balance	16–18	10–14	2–3	≤2	≤1	≤0.1	≤0.045	≤0.03	≤0.03

**Table 2:** Chemical composition of SS 316L stainless steel [32].

Element	Fe	Cr	Ni	Mo	Mn	Si	N	O	P	C	S
Mass (%)	Balance	16–18	10–14	2–3	≤2	≤1	≤0.1	≤0.1	≤0.045	≤0.03	≤0.03

**Table 3:** Chemical composition of M300 maraging steel [33].

Element	Fe	Ni	Co	Mo	Ti	Si	Mn	C	P	S
Mass (%)	Balance	17–19	7–10	4.5–5.2	0.3–1.2	≤0.1	≤0.15	≤0.03	≤0.01	≤0.01

**Table 4:** Mechanical and physical properties of the experimental materials [31,32,33].

Parameter	Direction	AISI 316	SS 316L (As Built)	M300 (As Built)
Density [g·cm^−3^]	-	8.03	7.99	8.10
Hardness [HV]	XY	187	198 ± 8 *	350 ± 15 *
	Z		208 ± 6 *	357 ± 12 *
Yield strength [MPa]	XY	205	547 ± 3	976 ± 17
	Z		494 ± 14	794 ± 22
Elongation at break [%]	XY	40	43 ± 2	15 ± 1
	Z		35 ± 8	10 ± 2
Modulus of elasticity [GPa]	XY	193	197 ± 4	185 ± 9
	Z		190 ± 10	189 ± 6
Thermal conductivity [W·mK^−1^]	-	16.3	16.2	14.2
Melting point [°C]	-	1375–1400	1371–1399	1413

* Vickers hardness HV0.5.

**Table 6:** EBW technological parameters.

Group	Specimen (Weld)	Materials	Welding Current (I_w_) [mA]	Welding Velocity (v_w_) [mm.s^−1^]
1	W1	AISI 316–AISI 316	135	35
	W2	AISI 316–AISI 316	135	30
	W3	AISI 316–AISI 316	135	25
	W4	AISI 316–AISI 316	150	20
2	W5	AISI 316–SS 316L	135	25
	W6	AISI 316–SS 316L	150	30
3	W7	M300–AISI 316	135	25
	W8	M300–AISI 316	150	20
	W9	M300–AISI 316	150	20
	W10	M300–AISI 316	150	25

## Data Availability

Data are contained within the article.
